# DeepGOPlus: improved protein function prediction from sequence

**DOI:** 10.1093/bioinformatics/btaa763

**Published:** 2021-05-03

**Authors:** Maxat Kulmanov, Robert Hoehndorf

*Bioinformatics* (2020) doi: 10.1093/bioinformatics/btz595

In the above article, the image currently appearing in the "Figure 2" position belongs instead in the "Figure 1" position; the image currently appearing in the "Figure 3" position belongs instead in the "Figure 2" position; the image currently appearing in the "Figure 1" position belongs instead in the "Figure 3" position. The publisher apologises for the error.

