# Supplementary material for: DeepGOPlus: improved protein function prediction from sequence
Source: Bioinformatics. 2019 Jul 27;36(2):422–9. doi: 10.1093/bioinformatics/btz595 (PMC9883727; doi:10.1093/bioinformatics/btz595)
Supplement: btz595_Supplementary_Data [file btz595_supplementary_data.pdf]

Supplementary materials: DeepGOPlus: Improved protein function prediction from sequence

April 18, 2019

#	MaxKernel	Hidden Layers	Filters	Valid. Loss	Test Loss
1	32	1	32	0.04085	0.024466
2	64	1	32	0.04032	0.024393
3	128	1	32	0.04038	0.024314
4	256	1	32	0.04008	0.024610
5	512	1	32	0.04019	0.025172
6	32	2	32	0.04187	0.024399
7	64	2	32	0.04194	0.024147
8	128	2	32	0.04125	0.026296
9	256	2	32	0.04054	0.025792
10	512	2	32	0.04134	0.024686
11	32	3	32	0.04173	0.024688
12	64	3	32	0.04215	0.025352
13	128	3	32	0.04110	0.025408
14	256	3	32	0.04109	0.024851
15	512	3	32	0.04104	0.026999
16	32	1	64	0.04028	0.023811
17	64	1	64	0.04016	0.024081
18	128	1	64	0.04024	0.024422
19	256	1	64	0.03986	0.025013
20	512	1	64	0.04039	0.024882
21	32	2	64	0.04169	0.024334
22	64	2	64	0.04117	0.025407
23	128	2	64	0.04080	0.026773
24	256	2	64	0.04043	0.025632
25	512	2	64	0.04039	0.026199
26	32	3	64	0.04160	0.024624
27	64	3	64	0.04142	0.026038
28	128	3	64	0.04029	0.025585
29	256	3	64	0.04016	0.025938

30	512	3	64	0.04052	0.026569
31	32	1	128	0.03996	0.023886
32	64	1	128	0.03999	0.024146
33	128	1	128	0.03990	0.024231
34	256	1	128	0.03945	0.025394
35	512	1	128	0.04002	0.024113
36	32	2	128	0.04131	0.024404
37	64	2	128	0.04116	0.024885
38	128	2	128	0.04000	0.026053
39	256	2	128	0.04000	0.026025
40	512	2	128	0.04037	0.023884
41	32	3	128	0.04100	0.025189
42	64	3	128	0.04031	0.025051
43	128	3	128	0.03995	0.025764
44	256	3	128	0.04135	0.024185
45	512	3	128	0.04008	0.024312
46	32	1	256	0.03942	0.023801
47	64	1	256	0.03933	0.024252
48	128	1	256	0.03897	0.024517
49	256	1	256	0.03950	0.024034
50	512	1	256	0.04146	0.024329
51	32	2	256	0.04050	0.024755
52	64	2	256	0.04026	0.025917
53	128	2	256	0.03992	0.025778
54	256	2	256	0.03995	0.025696
55	512	2	256	0.04071	0.025106
56	32	3	256	0.04129	0.025048
57	64	3	256	0.03997	0.024811
58	128	3	256	0.03918	0.025124
59	256	3	256	0.03920	0.026214
60	512	3	256	0.04045	0.026019
61	32	1	512	0.03912	0.024001
62	64	1	512	0.03881	0.024483
63	128	1	512	**0.03880**	0.024031
64	256	1	512	0.03966	0.024428
65	512	1	512	0.04201	0.024437
66	32	2	512	0.03929	0.025065
67	64	2	512	0.03913	0.025310
68	128	2	512	0.03945	0.024313
69	256	2	512	0.03954	0.024128
70	512	2	512	0.04030	0.024147
71	32	3	512	0.03947	**0.023562**
72	64	3	512	0.03925	0.025331
73	128	3	512	0.03902	0.024775
74	256	3	512	0.03969	0.024065
75	512	3	512	0.03971	0.025466

Table 1: Different parameters used to tune the model
